# Pragmatic Application of the RE-AIM Framework to Evaluate the Implementation of Tobacco Cessation Programs Within NCI-Designated Cancer Centers

**DOI:** 10.3389/fpubh.2020.00221

**Published:** 2020-06-12

**Authors:** Heather D'Angelo, Alex T. Ramsey, Betsy Rolland, Li-Shiun Chen, Steven L. Bernstein, Lisa M. Fucito, Monica Webb Hooper, Robert Adsit, Danielle Pauk, Marika S. Rosenblum, Paul M. Cinciripini, Anne Joseph, Jamie S. Ostroff, Graham W. Warren, Michael C. Fiore, Timothy B. Baker

**Affiliations:** ^1^Carbone Cancer Center, School of Medicine and Public Health, University of Wisconsin-Madison, Madison, WI, United States; ^2^Department of Psychiatry, Washington University School of Medicine, St. Louis, MO, United States; ^3^Alvin J. Siteman Cancer Center, Barnes-Jewish Hospital and Washington University School of Medicine, St. Louis, MO, United States; ^4^Institute for Clinical and Translational Research, School of Medicine and Public Health, University of Wisconsin-Madison, Madison, WI, United States; ^5^Yale Department of Emergency Medicine, Yale School of Medicine, New Haven, CT, United States; ^6^Department of Psychiatry, Yale School of Medicine, New Haven, CT, United States; ^7^Yale Cancer Center, Smilow Cancer Hospital at Yale-New Haven, New Haven, CT, United States; ^8^Case Comprehensive Cancer Center, Case Western Reserve University, Cleveland, OH, United States; ^9^Department of Medicine, Center for Tobacco Research and Intervention, School of Medicine and Public Health, University of Wisconsin-Madison, Madison, WI, United States; ^10^Department of Research, William S. Middleton Memorial Veterans Hospital, Madison, WI, United States; ^11^Department of Behavioral Sciences, University of Texas MD Anderson Cancer Center, Houston, TX, United States; ^12^Department of Medicine, Masonic Cancer Center, University of Minnesota, Minneapolis, MN, United States; ^13^Department of Psychiatry and Behavioral Sciences, Memorial Sloan Kettering Cancer Center, New York, NY, United States; ^14^Department of Radiation Oncology, Hollings Cancer Center, Medical University of South Carolina, Charleston, SC, United States; ^15^Department of Cell and Molecular, Pharmacology and Experimental Therapeutics, Hollings Cancer Center, Medical University of South Carolina, Charleston, SC, United States

**Keywords:** Tobacco treatment, Smoking Cessation, Cancer center, RE-AIM (Reach, Effectiveness, Adoption, Implementation and Maintenance), implementation

## Abstract

Tobacco cessation after cancer diagnosis leads to better patient outcomes. However, tobacco treatment services are frequently unavailable in cancer care settings, and multilevel implementation challenges can impede uptake of new programs. The National Cancer Institute (NCI) dedicated Cancer Moonshot funding through the Cancer Center Cessation Initiative (C3I) for NCI-Designated Cancer Centers to implement or enhance the implementation of tobacco treatment services. We examined a pragmatic application of the RE-AIM framework (reach, effectiveness, adoption, implementation, and maintenance) to evaluate tobacco treatment programs implemented within Cancer Centers funded through C3I. Using three C3I-funded Centers as examples, we describe how each RE-AIM construct was operationalized to evaluate the implementation of a wide range of cessation services (e.g., tobacco use screening, counseling, Quitline referral, pharmacotherapy) in this heterogeneous group of cancer care settings. We discuss the practical challenges encountered in assessing RE-AIM constructs in real world situations, including using the electronic health record (EHR) to aid in assessment. Reach and effectiveness evaluation required that Centers define the setting(s) where cessation services were implemented (to determine the “denominator”), enumerate the patient population, report current patient tobacco use, patient engagement in tobacco treatment, and 6-month cessation outcomes. To reduce site heterogeneity, increase data accuracy, and reduce burden, reach was frequently captured via standardized EHR enhancements that improved the identification of current smokers and tobacco treatment referrals. Effectiveness was determined by cessation outcomes (30-day point prevalence abstinence at 6-months post-engagement) assessed through a variety of data collection approaches. Adoption was measured by the characteristics and proportion of targeted cancer care settings and clinicians engaged in cessation service delivery. Implementation was assessed by examining the delivery of tobacco screening assessments and intervention components across sites, and provider-level implementation consistency. Maintenance assessments identified whether tobacco treatment services continued in the setting after implementation and documented the sustainability plan and organizational commitment to continued delivery. In sum, this paper demonstrates a pragmatic approach to using RE-AIM as an evaluation framework that yields relevant outcomes on common implementation metrics across widely differing tobacco treatment approaches and settings.

## Introduction

Continued smoking after a cancer diagnosis has been associated with adverse outcomes, including overall and cancer specific mortality, and increased risk of developing a second primary cancer. Importantly, smoking cessation after a cancer diagnosis improves clinical outcomes (1, 2). Several national cancer organizations have developed recommendations for integrating tobacco treatment as a routine component of cancer care (3–6). All patients seen in cancer care should be consistently assessed for tobacco use, and if they are current users (usually past 30 day use), should be advised to quit, and/or referred to cessation treatment (6, 7). While the recommendations are clear for *what* to implement, there is little published on *how* to implement and evaluate tobacco treatment programs in cancer care settings (7). Despite the availability of the National Comprehensive Cancer Network (NCCN) (6) and other Clinical Practice Guidelines for Smoking Cessation, tobacco treatment services are often not a routine component of cancer care (8–10).

To address this gap in research-to-practice, the National Cancer Institute (NCI) dedicated Cancer Moonshot funding to enhance the capacity of NCI-Designated Comprehensive Cancer Centers to implement sustainable evidence-based tobacco treatment programs (11). The resulting Cancer Center Cessation Initiative (C3I) funded 42 NCI-Designated Comprehensive Cancer Centers (“Centers”) to integrate tobacco treatment into cancer care. The C3I provides a unique opportunity to examine how tobacco treatment can be effectively implemented in cancer care. This study uses the RE-AIM framework (12) to evaluate how different tobacco treatment programs were implemented in diverse real-world clinical settings receiving the same level of supplemental funding (11).

RE-AIM has been used previously to evaluate tobacco treatment programs in healthcare settings (13, 14). However some elements of the framework, namely adoption, implementation, and maintenance, are often not reported (15, 16), and most published studies report on measures collected as part of a research study rather than a pragmatic application in multiple, diverse clinical settings. This paper provides examples of a pragmatic application of the RE-AIM framework to evaluate the implementation of real-world tobacco treatment programs in cancer care settings using simple, low burden measures easily gathered across clinical settings using electronic health records (EHRs) to aid in measurement (12, 17).

## Cancer Center Cessation Initiative Pragmatic Re-aim Application

The C3I funded 42 Cancer Centers from 28 states and the District of Columbia for 2 years to implement evidence-based tobacco treatment programs through a supplement to the Cancer Center Support Grant (11). The C3I Coordinating Center provides scientific and technical assistance to help Centers integrate tobacco treatment services into clinical care. The goals were to: (1) achieve consistent tobacco use screening and documentation for every patient; and (2) deliver evidence-based tobacco treatment to current smokers, ideally using the EHR to streamline referrals. Centers were free to choose evidence-based intervention components for their sites, including referrals to internal (e.g., counseling, medication) and external (e.g., Quitline) programs.

**Figure 1:**
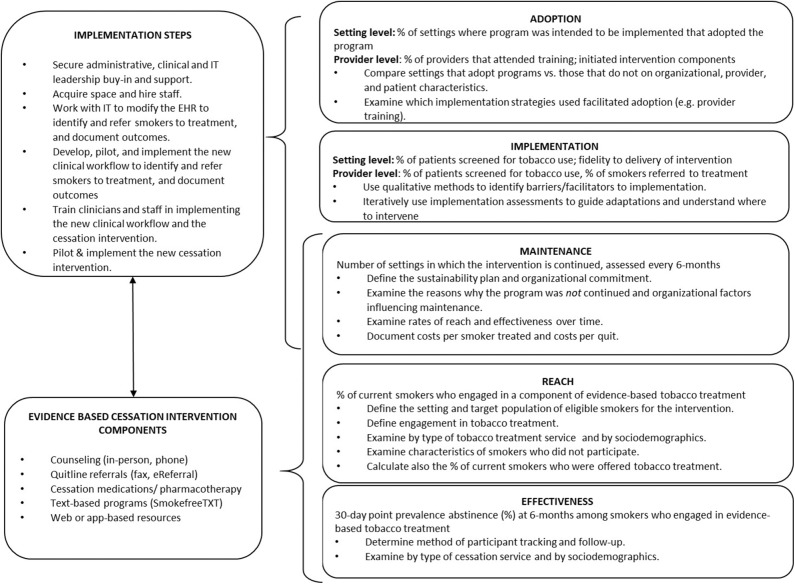
RE-AIM application to evaluate tobacco treatment program implementation in cancer care, and the related implementation steps and evidence-based intervention components employed.

The C3I Coordinating Center, in collaboration with an expert panel of physicians, psychologists, and behavioral scientists with clinical and implementation expertise in smoking cessation for cancer patients, developed measures to evaluate progress amongst the C3I Centers. Measures were drawn from a pragmatic application of the RE-AIM framework and intended to be low burden, actionable, sensitive to change, and broadly applicable to diverse cancer care settings (12). Figure 1 shows the RE-AIM application to evaluate tobacco treatment program implementation, the evidence-based program components implemented, and the related implementation steps employed. Measures were designed to be compatible with existing EHR functionalityto generate data for evaluation. The Coordinating Center facilitated the sharing of best practices across C3I sites, and created a learning environment where sites meet every 6 months to discuss their successes and challenges, including reporting on the evaluation of their program implementation. Coordinating Center recommendations to the C3I Centers for how each RE-AIM measure might be interpreted and used is presented below.

### Reach

Reach in C3I is defined as the proportion of current smokers seen in a cancer care setting who engaged or participated in an evidence-based tobacco treatment program. However, calculating reach is dependent upon the consistent identification of current smokers in the EHR (i.e., the denominator for reach) and upon the definition and documentation of patient engagement in an evidence-based tobacco treatment program (i.e., the numerator for reach). The following steps were offered to guide the assessment of reach by using the EHR to determine the numerator and denominator for the reach of tobacco treatment programs in cancer care settings. Table 1 shows the results for three NCI-Designated Cancer Centers in C3I.

1. Define the setting where patients are assessed for tobacco use and identified as current smokers during their medical visit (e.g., the whole cancer center, or certain clinics).
2. Count unique patients seen in the setting during a specific period (e.g., 6 months). Each Center determined the type of visit in which tobacco use assessment would occur, such as during registration or nursing assessment, and were encouraged to include patients seen for cancer screening or treatment. The EHR reporting team may need to set up filters for selecting patient encounters and/or rules for counting visits. The aim is to select visits with clinician-patient interactions where tobacco use assessment and referrals should occur for current smokers.
3. Count the number of patients screened for tobacco use to determine the tobacco use assessment rate (number of patients screened/total number of patients).
4. Determine the number of current smokers by counting patients with a current smoking status. In the EHR a current smoking status could include: current every day smoker; current some days smoker; heavy smoker; or light smoker, but may vary depending on how the EHR is programmed. This number serves as the *denominator* for reach.
5. Among current smokers, count the number who engaged in at least one type of evidence-based tobacco treatment, and the number who engaged in each type of treatment offered at the Center. This serves as the *numerator* for reach. Each Center defined engagement depending on the services offered and following these guidelines for what constitutes engagement:
  1. counseling (in-person, phone, including brief advice to quit),
  2. connection to a Quitline, web-based, or text/mobile program via fax or eReferral, or
  3. cessation medications prescribed.
  If a program counts *acceptance* to receive treatment (e.g., to be referred to a Quitline) as engagement, reach should be defined as such. The number of smokers who were *offered* a program should be recorded separately from those who did engage. This could include the number of smokers who were offered enrollment in a counseling program (regardless of engagement), or the number who were given educational materials but were not connected with a program. The number of current smokers who declined to participate should be documented as a target for quality improvement.
6. Wherever possible, each Center should record patient demographics for current smokers and program participants to determine the representativeness of those reached. Many EHRs capture data on patient gender, race, ethnicity, age, and primary insurance type.

**Table 1:** Description of tobacco treatment programs at three NCI-Designated Cancer Centers funded through the Cancer Center Cessation Initiative.

	**Washington University**	**Yale University**	**Case Western Reserve University**
Setting (*s*)	Siteman Cancer Center, St. Louis, MO	Smilow Cancer Hospital, New Haven, CT and Smilow Cancer Care Centers throughout CT	University Hospitals Seidman Cancer Center, MetroHealth Cancer Center, Cleveland Clinic Taussig Cancer Center, Cleveland, OH^b^
Patients with visits to the setting^a^, *N*	27,728	43,264	41,405
Patients screened for tobacco use^a^, *N*	25,779	21,424	32,541
Patients identified as current smokers^a^, *N*	3,224	3,882	4,316
Current smokers who engaged in at least one type of evidence-based cessation treatment^a^, *N*	1,390	277	907
Tobacco treatment program components	ELEVATE (Electronic Health Record-Enabled Evidence-Based Smoking Cessation Treatment) • Deliver smoking cessation counseling (5A's) and pharmacotherapy at the point of care. • Enhance the EHR to identify and refer current smokers to the Quitline and SmokefreeTXT. • Training and video-based demonstrations and simulated patient scenarios with clinical care providers using test patients in the EHR. • Monthly provider performance data feedback, in comparison to department- and/or clinic-level data and clinical benchmarks.	Tobacco Treatment Service (TTS) at Smilow Cancer Hospital • In-person counseling program including medication management, and training providers. • Phone/tele-health counseling also delivered. • Smokers are also referred to SmokefreeTXT. • Monthly audit and feedback reports on Care Center performance are prepared and reviewed by the Tobacco Treatment Service and shared with Smilow Cancer Hospital and Care Center leadership.	Tobacco Intervention & Psychosocial Support (TIPS) Service • Face-to-face cognitive behavioral therapy combined with pharmacotherapy. • Tailored to cancer patients by including cancer-specific psychoeducation, emotional vulnerability content, and flexible intervention formats (e.g., in-clinic, telephone). • Caregivers and/or significant others may also be treated. • Smokers may also be referred to the SmokefreeTXT.
Smoker identification and referral method(s)	• Current smokers identified through the EHR during visit. • Patients with a current smoker status or documented as using a cessation medication will trigger a BPA that prompts the clinician to deliver counseling and pharmacotherapy at the point-of-care. • Referrals to the Quitline and SmokefreeTXT generated through the EHR.	• Current smokers identified through the EHR. • A list of current smokers sent to program staff who contact patients to schedule appointments. • eReferral to SmokefreeTXT is in development.	• Current smokers identified through the EHR during visit. Patients who indicate motivation to quit are referred via email, pager, or through eReferral (in select clinics). • Patients are contacted by TIPS staff for the initial assessment, treatment planning, and schedule for counseling appointments. • Patients may be signed up for SmokefreeTXT by program staff or may self-enroll. • Caregivers/significant others may be seen with the patient or contacted independently by program staff
EHR modifications implemented	Developed new clinical workflow, BPAs, and eReferral systems.	• Modified clinical workflow. • Enhanced the EHR to standardize tobacco use assessment. • eReferral sends prompt to Tobacco Treatment Service for current smokers.	• Standardized tobacco use assessments based on NCCN guidelines. • EHR provider notes generated to summarize tobacco treatment services delivered. • EHR eReferral generated to send patient information to TIPS

a *Reported for a 6-months period at 1 year post-implementation*.

b *Sum across the three healthcare systems*.

### Effectiveness

Effectiveness is assessed by examining quit rates among those who participate in cessation treatment. In C3I, outcomes are assessed at 6-months post-engagement with one item; “When did you last smoke a cigarette (even one or two puffs)?,” which allows for the calculation of both 7- and 30-day point prevalence abstinence rates. Documentation may occur in a separate database or within the EHR, although this may require additional programming. Follow-up assessments can be conducted in-person, via telephone, or through Quitline reports. In line with reach, effectiveness should be examined by patient sociodemographics and the type of tobacco treatment program used to explore variation in cessation outcomes.

### Adoption

Setting level adoption is defined as the proportion of settings targeted for implementation that initiated the program. Adoption can be examined by organizational characteristics to understand local barriers. For example, are the administrative leaders in some settings hesitant to make changes to the EHR, or is there high staff turnover that makes clinical leadership hesitant to devote time to staff training? Provider level adoption can be assessed by documenting participation in training and tracking initiation of program components. Examining which implementation steps facilitated adoption (e.g., securing buy-in, provider trainings) should suggest how tobacco treatment can be enhanced at the setting and provider level.

### Implementation

Level of implementation can be indexed by the quality and consistency of tobacco treatment service delivery. Examining provider level tobacco use screening, advice and referral rates can identify high- and low-performing providers, which can be used to focus additional training. Intervention fidelity can be assessed by examining the delivery of intervention components, such as brief advice, counseling sessions delivered as intended, and whether medications were prescribed. Such information can be used to understand the sources of variation in intervention effectiveness: (i.e., is it the intervention or is it the level of implementation?) Qualitative methods, such as stakeholder interviews with patients, clinicians, and administrators can be used to examine barriers to implementation.

### Maintenance

Maintenance can be defined as the degree to which rates of reach and effectiveness are sustained across time, as well as the potential for sustainability of the program. Defining the sustainability plan and securing organizational commitment to the program are key elements in estimating sustainability potential. After implementation, the number of settings in which the program is continued can be assessed, with a qualitative examination of the reasons programs were not maintained, with a comparison of maintained/not maintained settings on organizational, provider, and patient characteristics.

## Examples of Re-aim Application at C3I Cancer Centers

### Washington University

#### Context

The EHR-enabled Evidence-based Smoking Cessation Treatment (ELEVATE) program developed at Washington University was implemented at the Siteman Cancer Center, which serves about 25,000 patients per year across rural and urban areas and medically underserved populations in multiple Midwestern states. The implementation of ELEVATE coincided with the launch of a new EHR platform at Washington University and BJC Healthcare system. ELEVATE leverages newly developed EHR modifications, including enhanced clinical workflows, Best Practice Alerts (BPAs), and automated referral systems to prescribe smoking cessation medications and provide counseling resources at the point-of-care (Table 1). Patients with a status of current smoker or a prescription for cessation medication are defined as “current smokers,” which triggers a BPA prompting the clinician to deliver brief advice, prescribe medications, and refer patients to “light-touch” resources including the Quitline, SmokefreeTXT, and Smokefree smartphone apps.

The ELEVATE program is supported by a bundled implementation strategy that includes: formal and informal training exercises through in-person and video-based demonstrations of ELEVATE module use, technical assistance and recommendations following live patient encounters and simulated patient scenarios with clinical care providers using test patients in the EHR, and monthly performance data feedback delivered to medical assistants and physicians that provides data on assessment and treatment rates at the provider level and in comparison to department- and/or clinic-level data and clinical benchmarks. With the emphasis on data-driven quality improvement, provider-level assessment and treatment rates are expected to increase over time.

#### Reach

Smoking prevalence was 12.5% among patients screened for tobacco use who had at least one outpatient oncology clinic visit within a 6-month period (Table 2). Reach was defined as the proportion of current smokers who received either of the following types of tobacco treatment documented in the EHR within a 6-month period: active smoking cessation medication prescribed (i.e., medication rate) or brief cessation counseling (i.e., brief counseling rate). Before ELEVATE implementation, overall reach was 3.6%; after implementation, reach increased to 43.1%. Interest in counseling (i.e., counseling offer rate), and referral to phone-based, SMS text-based, and/or smartphone app-based counseling (i.e., counseling referral rate) were recorded separately from engagement.

**Table 2:** Pragmatic application of RE-AIM to evaluate tobacco treatment program implementation at three NCI-Designated Cancer Centers funded through the Cancer Center Cessation Initiative.

**RE-AIM construct**	**Evaluation measures**^****a****^		**Washington University Siteman Cancer Center**	**Yale University Smilow Cancer Hospital**	**Case Western Reserve University Case Comprehensive Cancer Center^**c**^**
REACH	Smoking prevalence^b^		12.5%	18%	21.1%
	Smokers reached with at least one evidence-based cessation treatment		43.1% of smokers were prescribed cessation medications and/or received brief counseling at the point-of-care	7% of smokers were prescribed cessation medications, referred to the TTS, and/or referred to SmokefreeTXT	24.3% of smokers were prescribed cessation medications, referred to TIPS, and/or referred to SmokefreeTXT
EFFECTIVENESS	Assessment method		Tobacco use status from EHR for most recent visit during 6-months period post-treatment	Assessed at 6-months in person or via phone & documented in EHR	Assessed at 6-months in person or via phone and documented in EHR.
	6-month follow-up rate		67.2%	13.5%	54.4%
	30-day point prevalence abstinence	Counting patients lost to follow-up as smokers	29.5%	2.2%	19.5%,
		Among patients with follow-up data	43.9%	16.7%	35.1%
ADOPTION	Setting level adoption		21/21 outpatient oncology clinics over a 6-months implementation period.	Adopted at Smilow Cancer Center and 9/10 Care Centers over ~8 months.	Adopted in 3/3 healthcare systems. One launched center-wide, two launched in thoracic and gynecological oncology clinics.
	Provider level adoption		99% providers initiated assessment, 79% initiated documentation of medication, 85% initiated offer of counseling referral.	Not assessed	Number of referring providers (*N* = 64) has increased by 25% over 1 year of implementation.
IMPLEMENTATION	Setting level tobacco use assessment rate		93%	49.5%	80%
	Provider-level tobacco use assessment rate		93% providers achieved ≥90% rate	Not assessed	Not assessed
	Implementation of key program components		Pharmacotherapy rate: 49% of providers achieve ≥20% rate; Counseling offer rate: 51% of providers achieve ≥50% rate	BPA utilization rates for referrals to the TTS, pharmacotherapy or both.	51% of referred patients received at least one component of the TIPS intervention.
MAINTENANCE	Sustainability plans/goals		• 6-months ongoing reach and effectiveness evaluation. • Incentivize care using data transparency and performance feedback. • Low-burden, low cost decision support tool for point-of-care use ($3 per patient).	Hiring another tobacco treatment specialist to maintain program at Care Centers. Billing for services using an APRN and expanding telehealth services. Integrating referrals into new patient onboarding by nurse navigators.	• Leverage initial success. • Generate new funding sources. • Reduce patient barriers to treatment (e.g. cost, transportation. • Identify 100% of smokers & reach at least 50% of smokers with treatment.

a *Reported for a 6-month period at 1-year post-implementation*.

b *Among patients screened for tobacco use*.

c *Average of three cancer healthcare settings*.

*TTS, Tobacco Treatment Service; TIPS, Tobacco Intervention & Psychosocial Support*.

#### Effectiveness

The EHR was used to assess current smoking status at 6-months post-tobacco treatment. This method relied on patients having an updated tobacco use status at 6-months after receiving tobacco treatment. Using this method, 67.2% of patients that received tobacco treatment had follow-up data available in the EHR in the following 6 months. Before ELEVATE was implemented, EHR data indicated that only 2.3% of patients treated for smoking had not smoked in the past 30 days at 6-months post-treatment. Following ELEVATE, 43.9% of smokers who received brief counseling, medications, or both and who had 6-month follow-up data documented in the EHR had not smoked in the past 30 days (29.5% using an intent to treat principle counting those lost to follow-up as current smokers). In contrast, only 7.6% of smokers who did *not* receive tobacco treatment reported they were no longer current smokers 6-months following their cancer center visit.

#### Adoption

At the setting level, all 21 outpatient oncology clinics in the Siteman Cancer Center adopted ELEVATE and initiated tobacco assessment and treatment services with the new point-of-care EHR module. At the provider level, at 1-year post-implementation, EHR data revealed that 99% of providers/clinic staff had initiated use of the new smoking status assessment, 79% initiated medication documentation, and 85% initiated the counseling referral components of ELEVATE, indicating high levels of adoption.

#### Implementation

The tobacco use assessment rate was 93% over a 6-month period 1 year after implementation. In contrast, the assessment rate was only 47.9% in the 5 months preceding the ELEVATE launch (18). Provider-level rates of assessment and treatment varied substantially. Over a 6 month period, 93% of medical assistants documented tobacco use assessment for at least 90% of patient encounters. During this time period, 51% of providers offered a counseling referral during at least half of their patient encounters.

#### Maintenance

Longer-term data on reach and effectiveness will be collected every 6-months. Sustainability is often driven by a favorable “implementation climate,” characterized by the extent to which delivering tobacco treatment is expected, supported, and rewarded. We believe the training strategies, data transparency, and performance feedback will enhance maintenance, as will the tactical design of ELEVATE as a low-burden point-of-care decision support tool. The program utilizes an embedded cancer care team, with no plans to hire tobacco treatment specialists or additional staff. As a result, there are no discrete costs for dedicated personnel, and the cost per patient is $3, which promotes sustainability of the program.

### Yale University

#### Context

The Tobacco Treatment Service (TTS) at Smilow Cancer Hospital in New Haven, CT was started in 2011 and expanded in 2017 after receiving C3I funding to include several Cancer Care Centers across Connecticut (Table 1). The TTS offers smoking cessation counseling (in-person, televideo, or phone-based), medication management, and referrals to the NCI SmokefreeTXT program. EHR modifications improved the identification and treatment of current smokers. Streamlining the EHR tobacco use assessment section was proposed, but would have required changes across the whole health system and therefore was not accepted at the organizational level. Due to this barrier, efforts were redirected toward revising the BPA to increase utilization. The previously existing BPA required multiple steps on the part of the provider and ordered a TTS referral only. Through feedback from providers and beta testing, the BPA was optimized to be less disruptive to clinical workflows, include all necessary steps and documentation in one click (i.e., diagnosis, after visit summary, smoking counseling note, and CPT billing code), and include the option to order tobacco pharmacotherapies (i.e., varenicline or nicotine patches and lozenges) using pre-populated fields based on the patient's current tobacco use.

#### Reach

EHR generated reports documented the number of current smokers, and the number who were referred to the TTS and/or prescribed cessation medication. The TTS received notification of patients referred to SmokefreeTXT via a separate reporting mechanism. Over a 6-month period after program implementation, smoking prevalence among those screened was 18%. Among the documented current smokers, reach for the Smilow Cancer Hospital and the Care Centers over 6 months was 7% (Table 2). Among those reached, 58.5% received in-person counseling, and 97.4% received medications.

#### Effectiveness

Participants who receive counseling from the TTS are offered follow-up visits after 6 months to assess and document current smoking status in the EHR. Follow-up can be challenging because some patients withdraw from the TTS, appointment availability may not always align with patients' schedules, and there are limited resources to maintain contact. Among those who completed 6-month follow-up visits (*n* = 30 out of 223 participants), the 30-day point prevalence abstinence rate was 16.7% (2.2% using an intent to treat principle counting those lost to follow-up as current smokers) (Table 2).

#### Adoption

Programmatic adoption occurred in stages. The TTS program has been adopted at nine of 10 Smilow Cancer Care Settings in addition to the Smilow Cancer Hospital. One site declined to participate due to an established relationship with a smoking cessation program at another local hospital. Care Centers were visited by TTS staff to establish relationships with clinical and administrative staff, which facilitated the adoption of the new clinical workflow. Adoption occurred over about 8 months, with two to three Care Centers added every 1 to 2 months. This gradual expansion allowed for piloting the BPA first in a few sites, revealing a need to modify the EHR to allow for referrals from RNs in addition to MDs and Advanced Practice Providers, and a need for educational meetings with nursing staff. A TTS “Champion” partner was identified at each Care Center to help integrate services into the center.

#### Implementation

Rates of tobacco use assessment and documentation are examined by clinical setting and by provider. The average tobacco use assessment rate for a 6-month period was 49.5% across settings, primarily because tobacco use assessment in the EHR is not mandatory. Reports are generated to show: (1) the number of times the BPA “fired,” or appeared to a provider (2) the number of times the BPA “fired” and was acted on, and (3) the number of acted on BPA fires that included a referral to the TTS only, tobacco pharmacotherapy orders, or both. The data are then used to identify settings or providers with lower BPA utilization rates to provide feedback and troubleshoot barriers. For example, at one Care Center, low utilization was due to limited staffing following the departure of an oncologist. The remaining clinical staff were unable to devote substantial resources to implementing enhanced care for their patients who smoked, because their patient loads had increased.

#### Maintenance

Currently, one staff member provides counseling services at nine Care Centers on a rotating weekly schedule, traveling up to 900 miles per month. To maintain the program and increase capacity for treatment provided at each site, another full-time APRN was hired. As NCI grant funding comes to an end, Smilow Cancer Hospital will take over funding for the TTS providers, who will eventually bill for services. Additional maintenance efforts include expanding telehealth options and working with nurse navigators who onboard new cancer patients to integrate the TTS into the standard treatment offered at Smilow.

### Case Western Reserve University

#### Context

Case Comprehensive Cancer Center (Case CCC) serves 15 counties in Northeast Ohio. In Cleveland, the most populous city in the catchment area, smoking prevalence (35%) and lung cancer mortality rates exceed national averages. Case CCC consists of Case Western Reserve University School of Medicine, University Hospitals Seidman Cancer Center (SCC), Cleveland Clinic Taussig Cancer Institute (TCI), and is closely affiliated with the county safety-net hospital, MetroHealth Cancer Center (MHCC). Together, these cancer centers see about 20,000 new cancer cases annually and comprise a complex clinical setting in which to implement change. Each health system sees a distinct patient population, with underserved and racial/ethnic minority cancer patients seen largely at MHCC. In 2017, the Tobacco Intervention and Psychosocial Support (TIPS) Service was implemented in all three health systems and was designed to address the unique needs of cancer patients and survivors.

Clinicians screen for tobacco use and identify current smokers using the EHR during new patient visits. The tobacco use assessment questions were standardized across the three healthcare settings based on NCCN guidelines (6). The provider note includes a field to assess tobacco use status and tobacco use history/nicotine dependence. For current users, readiness to quit is assessed; relapse risk is assessed among recent quitters/former smokers. EHR modifications included programming to generate provider notes to summarize services delivered. Current tobacco users who indicated willingness to quit within the next 4 weeks are referred to TIPS either via an EHR-based order for counseling, or via email/pager. Irrespective of their willingness to participate in counseling, patients have the option to be enrolled in SmokefreeTXT. TIPS delivers cessation counseling using cognitive behavioral therapy combined with FDA-approved pharmacotherapy. TIPS is tailored to cancer patients by including cancer-specific psychoeducation, content that addresses the emotional vulnerability of this population, and flexible intervention formats (e.g., in-clinic, telephone, combination). Caregivers, family members, and/or significant others who use tobacco products are eligible for TIPS, and may participate with the patient, or independently.

#### Reach

Reach was defined as the proportion of current smokers who participated in TIPS, enrolled in SmokefreeTXT, and/or were prescribed cessation medication. Over 6 months, the average prevalence of current smoking was 21.1% among those screened, and an average of 24.3% of smokers across the three sites received at least one type of tobacco treatment (Table 2). Pharmacotherapy was the most common treatment type (82.1%), followed by in-person counseling (10.2%). Of note, 98% of patients who received counseling or another intervention also received pharmacotherapy.

#### Effectiveness

Effectiveness is assessed at 6-months after TIPS engagement. Program staff contact patients to document their current smoking status via telephone or interview patients in person if they have a scheduled an appointment. An average of 54.4% of TIPS participants were reached at 6-months follow-up across the three healthcare settings, after 1 year of implementation. Challenges to follow-up include patients being unreachable by phone or not being scheduled for a clinic follow-up near the time of assessment. Among TIPS patients with follow-up data, average 30-days abstinence at 6-month post-treatment was 35.1% across the three sites. Using an intent-to-treat principle (assuming patients lost to follow-up were still smoking) there was an average 30-day abstinence rate of 19.5%.

#### Adoption

The TIPS program was adopted in all three Case CCC affiliated health systems. Two health systems focused the initial implementation in thoracic and gynecological oncology clinics and the third opted for a center-wide launch. Pre-implementation program site leaders and clinical champions were instrumental to build clinical capacity, train staff, modify the EHR to standardize the assessment and documentation of tobacco use and treatment, and to integrate TIPS into the clinical workflow. Several implementation strategies facilitated the adoption of TIPS into the clinical workflow, including securing support from clinical and administrative leadership, operations staff, and IT specialists early in the process; seeking input from providers and staff during clinical division meetings and grand rounds; developing marketing strategies; and facilitating TIPS staff and medical team engagement. Initial provider engagement strategies have been encouraging, as the number of referring providers (*N* = 64) has increased by 25% over the past year with growing awareness of the service. Adoption challenges included securing adequate space, buy-in from providers with many competing responsibilities and limited time with patients, and the lengthy period to implement requested additions to the EHR.

#### Implementation

Implementation was assessed for the following elements key to delivering the TIPS program: tobacco use screening, provider referrals, and intervention delivery to smokers referred. The average rate of tobacco use assessment was 80%, which was negatively affected by EHR programming challenges at one of the hospitals (we anticipate the rate will increase). Over a 6-month period following implementation, 51.4% of patients referred to TIPS completed the tobacco history assessment and at least one intervention component of TIPS.

### Maintenance

TIPS service adoption is ongoing in three cancer hospitals that serve Northeast Ohio, the first regional effort to address tobacco use in cancer care settings. To maintain TIPS, the goal is to leverage the initial success of the effort, sustain EHR modifications to facilitate assessment and referrals, develop new strategies to increase provider and patient engagement, generate funding sources, and examine strategies to reduce patient-level barriers (e.g., cost/copays, transportation). The sustainability goals are to identify 100% of current tobacco smokers (and recent quitters), maintain an overall program reach of at least 50% of eligible patients, and demonstrate abstinence rates that are at least comparable to published estimates.

## Discussion

Using case studies from three funded C3I Cancer Centers, this report describes the application of the RE-AIM framework and the operationalization of each construct to evaluate the implementation of a range of cessation services (e.g., counseling, Quitline) in cancer care settings. The RE-AIM measures proposed have implications for cancer care settings beyond NCI Cancer Centers. The measures are flexible enough to work in different settings and for different types of tobacco treatment programs but are robust enough to measure intended evaluation outcomes. The measures can be applied across different healthcare systems and EHR platforms. C3I Centers largely used the funding to enhance the EHR to identify and refer smokers to treatment (19). As a result, the examples described provide guidance on using the EHR to assess RE-AIM constructs to evaluate the implementation of tobacco treatment programs integrated into cancer care. Each Center identified common challenges to measuring RE-AIM, or “lessons learned,” for other cancer care settings to be aware of when implementing tobacco treatment programs (Table 3).

**Table 3:** Summary of challenges to the measurement of RE-AIM within three NCI-Designated Cancer Centers funded through the Cancer Center Cessation Initiative.

**RE-AIM dimension**	**Challenges to measurement**
Reach	Measurement relies on consistent documentation of patient smoking status and engagement in tobacco treatment services.
Effectiveness	Follow-up measures (at 6-months post engagement) are dependent upon patient availability and program staff and resources to maintain follow-up contacts.
Adoption	Measuring provider-level adoption is dependent upon program and organizational resources to track and obtain provider-specific reports from the EHR.
Implementation	Measuring the implementation of key program components is dependent on program resources to document and produce reports using the EHR.
Maintenance	Measuring maintenance and sustainability is dependent on the program's ability to measure the other RE-AIM dimensions. Reporting on each of these measures over time can help Cancer Centers understand the long term sustainability of their program.

Previous work has shown that systems level changes, including EHR modifications, for assessing and referring patients to treatment can result in increased tobacco use documentation and counseling referrals (20–23). The profiled Centers utilized the EHR to identify and refer smokers to tobacco treatment services and to evaluate reach. Measuring reach posed challenges. Documenting the denominator (the number of current smokers) relies both on the consistent documentation of smoking status, and a way to extract that information from the EHR. Measuring the numerator for reach requires defining and documenting program engagement. At Washington University, the numerator included patients prescribed cessation medication or who had received counseling at the point-of-care. At Yale and Case, the reach numerator included patients who participated in in-person or telephone counseling, SmokefreeTXT, and/or were prescribed cessation medication. Because the treatment offered differs, defining the numerator is critical when making cross site comparisons. Reach is likely greater at Washington University because cessation counseling is delivered at the point-of-care, while the others refer to a counseling program.

Measuring effectiveness posed a different set of challenges (e.g., low rates of smoking status ascertainment), which may limit information regarding quit rates (20). While assessment of long-term smoking status is challenging, the EHR provides a highly efficient and cost-effective means of gathering follow-up data on patients receiving treatment. Each Center used the EHR to document tobacco use status at follow-up. However, Washington University utilized the EHR as the primary method to assess tobacco use with the patient's most recent visit at 6 months after tobacco treatment. The others used in-person or phone follow-up as the primary assessment. In reality, a combination of approaches may be necessary, where patients are contacted at follow-up to determine outcomes, but EHR-documented tobacco use status could be used when patients are not reached. Capturing the assessment of smoking outcomes in health system delivered programs is vital since reduced smoking prevalence is a key goal. Such data would yield meaningful outcomes upon which different implementation and intervention strategies can be compared.

In addition to reach and effectiveness, measuring implementation and adoption was facilitated by generating EHR reports for screening rates and provider-level program referrals. Two Centers provided monthly provider and clinic level performance data to show progress and identify areas for improvement. Non-adopting sites or providers may signal local barriers to initiation ranging from awareness, to self-perceived competence, to lack of supporting resources. Implementation strategies, such as staff training and practice facilitation, pairing non-adopting sites with mentor sites to share knowledge and resources, or identifying “champions” may be needed to address barriers and increase site- and provider-level adoption rates. Monthly data not only reflect adoption and implementation across providers and clinics, but also show trajectories that speak to maintenance. However, sites may be limited by organizational capacity to report back provider-level adoption and implementation metrics that may be useful for evaluation. The evaluation of provider level measures could be built into the EHR during program development, as making changes to the EHR after the fact is often challenging.

There are some limitations to this study. The RE-AIM application was examined within well-resourced Centers receiving funding to implement tobacco treatment services; however, it is unknown how readily less well-resourced cancer care settings without robust health informatics support could query the EHR to extract RE-AIM relevant data. The profiled Centers engaged in EHR modifications permitting efficient collection of evaluation measures, which may limit the adoption of this RE-AIM approach given significant resource requirements. Implementation at Washington University coincided with the launch of a new EHR platform allowing for more changes to the EHR than the other Centers. Data on reach and effectiveness were collected by patient sociodemographics; however, presenting this information was beyond the scope of this paper. Information on cost was not available, however cost data are being collected from C3I Centers and will be reported to inform program implementation in other cancer care settings. It is premature to report on the long-term maintenance of reach and effectiveness among programs overall and across different patient demographics. As programs mature, evaluation of demographics may facilitate the adoption of programs to better suit patient populations and is an important indicator of whether programs are equitably reaching all cancer patients who smoke.

Delivering tobacco treatment to cancer patients who smoke should be a routine and integrated part of cancer care (24). RE-AIM provides a framework for multilevel program evaluation to ensure patient benefit, provider performance, and organizational commitment. RE-AIM provides a vital component of an audit and feedback strategy by yielding performance data to inform normative comparisons, rewards, and encouragement to improve, along with existing resources and supports for the lower-performing groups. Conducting routine RE-AIM evaluations via the EHR allows program staff to rapidly identify gaps in care and address barriers with targeted strategies. A common RE-AIM approach to implementation assessment allows for trans-program comparisons to identify effective implementation and intervention strategies. The programs described provide tobacco treatment program staff working in cancer care settings with specific examples of measuring each RE-AIM dimension using the EHR to facilitate measurement. In summary, the measures demonstrate a pragmatic approach to using RE-AIM as an evaluation framework that yields relevant outcomes on common implementation metrics across widely differing tobacco treatment approaches and cancer care settings.

## Data Availability Statement

The datasets generated for this study are available on request to the corresponding author.

## Author Contributions

HD'A, BR, MF, and TB contributed to the conception and design of the study. HD'A wrote the first draft of the manuscript. AR, MW, LF, and SB wrote sections of the manuscript. All authors contributed to manuscript revision, read and approved the submitted version.

## Conflict of Interest

The authors declare that the research was conducted in the absence of any commercial or financial relationships that could be construed as a potential conflict of interest.
